# Precision immunoprofiling by image analysis and artificial intelligence

**DOI:** 10.1007/s00428-018-2485-z

**Published:** 2018-11-23

**Authors:** Viktor H. Koelzer, Korsuk Sirinukunwattana, Jens Rittscher, Kirsten D. Mertz

**Affiliations:** 1 Institute of Cancer and Genomic Science, University of Birmingham, 6 Mindelsohn Way, Birmingham, B15 2SY UK; 2 Molecular and Population Genetics Laboratory, Wellcome Centre for Human Genetics, University of Oxford, Headington, Oxford, OX3 7BN UK; 3 Institute of Biomedical Engineering, Department of Engineering Science, University of Oxford, Old Road Campus Research Building, Headington, Oxford, OX3 7DQ UK; 4 Ludwig Institute for Cancer Research, Nuffield Department of Medicine, University of Oxford, Old Road Campus Research Building, Oxford, OX3 7DQ UK; 5 Target Discovery Institute, NDM Research Building, University of Oxford, Old Road Campus, Headington, OX3 7FZ UK; 6 Institute of Pathology, Cantonal Hospital Baselland, Mühlemattstrasse 11, CH-4410 Liestal, Switzerland

**Keywords:** Personalized medicine, Immuno-oncology, Immunotherapy, Digital pathology, Image analysis, Machine learning, Artificial intelligence

## Abstract

Clinical success of immunotherapy is driving the need for new prognostic and predictive assays to inform patient selection and stratification. This requirement can be met by a combination of computational pathology and artificial intelligence. Here, we critically assess computational approaches supporting the development of a standardized methodology in the assessment of immune-oncology biomarkers, such as PD-L1 and immune cell infiltrates. We examine immunoprofiling through spatial analysis of tumor-immune cell interactions and multiplexing technologies as a predictor of patient response to cancer treatment. Further, we discuss how integrated bioinformatics can enable the amalgamation of complex morphological phenotypes with the multiomics datasets that drive precision medicine. We provide an outline to machine learning (ML) and artificial intelligence tools and illustrate fields of application in immune-oncology, such as pattern-recognition in large and complex datasets and deep learning approaches for survival analysis. Synergies of surgical pathology and computational analyses are expected to improve patient stratification in immuno-oncology. We propose that future clinical demands will be best met by (1) dedicated research at the interface of pathology and bioinformatics, supported by professional societies, and (2) the integration of data sciences and digital image analysis in the professional education of pathologists.

## Introduction

A picture is worth a thousand words. This is the essence of the technological transition from macropathology to micropathology as captured in Virchow’s core principle “omnis cellula et cellulae” first popularized in 1858 [81]. One hundred sixty years later, pathologists are in a technological transition phase of similar importance [61]: We are beginning to recognize that images of cells contain more information than what can be extracted by the human eye [20, 67, 83]. Computer-aided image analysis has the potential to make complex morphological information more accessible in daily diagnostic practice, improving prognostic and predictive patient stratification. Artificial intelligence has already been successfully employed in the setting of computational pathology to categorize diseases based on their molecular features [15, 21, 59]. In combination, it seems likely that image-based digital pathology in combination with artificial intelligence will become part of a pathologist’s tool repertoire in the near future.

Immuno-oncology requires a detailed understanding of the tumor microenvironment, including the identification and quantification of different immune cell subsets, their spatial context, and the expression of immune checkpoint markers. Changes in immune cell infiltration and biomarker expression before and after therapeutic intervention are critical parameters for clinical development [80]. Image analysis tools can carry out complex and repetitive biomarker analyses with high precision and excellent reproducibility and can thus greatly assist pathologists in their key role to integrate clinical, morphologic, and molecular information for personalized treatment [20, 83, 84]. Actively engaging in image analysis methods is therefore becoming increasingly important for pathologists to maintain their role as leaders in precision medicine and diagnostics.

In recent decades, electronic data processing has transformed medical radiology, molecular diagnostics, and genetic testing. At the same time, the practice of pathology which forms a crucial link between these clinical disciplines has not changed significantly. The current convergence of new imaging technologies with tissue-based multiplexed immunohistochemistry (IHC) (reviewed in “Multiplexing”) and molecular phenotyping, our ability to digitize and process large collections of histology slides, and the promise of supporting human interpretation through automated analysis and artificial intelligence will have a dramatic impact on the field [15, 59].

While traditional medical device companies, such as Philips, GE, and Leica advance new platforms for digital pathology and commercialize new imaging technologies, the major IT companies, including Google, IBM, and Microsoft, as well as numerous start-up companies (e.g., PathAI) enter the space by applying their expertise in big data science and artificial intelligence to data analysis and integrated decision making. Pharmaceutical companies have also recognized the importance of advanced pathology to their own work. Roche has already received FDA clearance for the VENTANA MMR IHC Panel for patients diagnosed with colorectal cancer (CRC) [2]. Developing and maintaining an understanding of digital imaging and data mining will therefore be beneficial skills for pathologists practicing in the twenty-first century [67, 83].

This paper provides a comprehensive outline of image analysis and machine learning (ML) applications for precision immunoprofiling. We will demonstrate how digital tools can facilitate pathology workflows in the assessment of established immune biomarkers and enable the deep characterization of the tumor microenvironment through spatial analysis and multiplexing. Further, we will identify computational methods driving morpho-molecular integration and deep learning methodologies for the discovery of novel therapeutic targets.

### Computational pathology to assist in the assessment of established biomarkers

Immuno-oncology has been revolutionized by the introduction of immune checkpoint inhibitors (ICI). ICI are monoclonal antibodies targeting immuno-regulatory molecules on the surface of T cells, antigen-presenting cells, and neoplastic cell populations [39]. Clinical success of reagents blocking the CTLA-4 (*cytotoxic T lymphocyte-associated protein 4*, *CD152*) and PD-1/PD-L1 checkpoints (*programmed cell death protein 1*, *CD279*; *programmed death-ligand 1*, *CD274*) has driven rapid regulatory approval for treatment of patients with both solid and hematologic malignancies [33]. Assessment of PD-L1 expression by IHC has emerged as an important predictive biomarker for patients with non-small cell lung cancer (NSCLC) [12, 13], urothelial carcinoma [34], and renal cell cancer [60]. However, assessment of PD-L1 is inherently difficult due to expression in both neoplastic and non-neoplastic cell populations, considerable marker heterogeneity and non-intuitive cutoffs. To complicate matters, PD-L1 positivity thresholds vary [80]. Inter-observer variability of PD-L1 assessment by pathologists is a known problem [17, 77]. This may contribute to inaccurate patient stratification and the misinterpretation of the impact of PD-L1 expression on clinical outcome: Subjective decisions can lead to radically different therapeutic stratification when scoring around cutoffs.

**Fig. 1:**
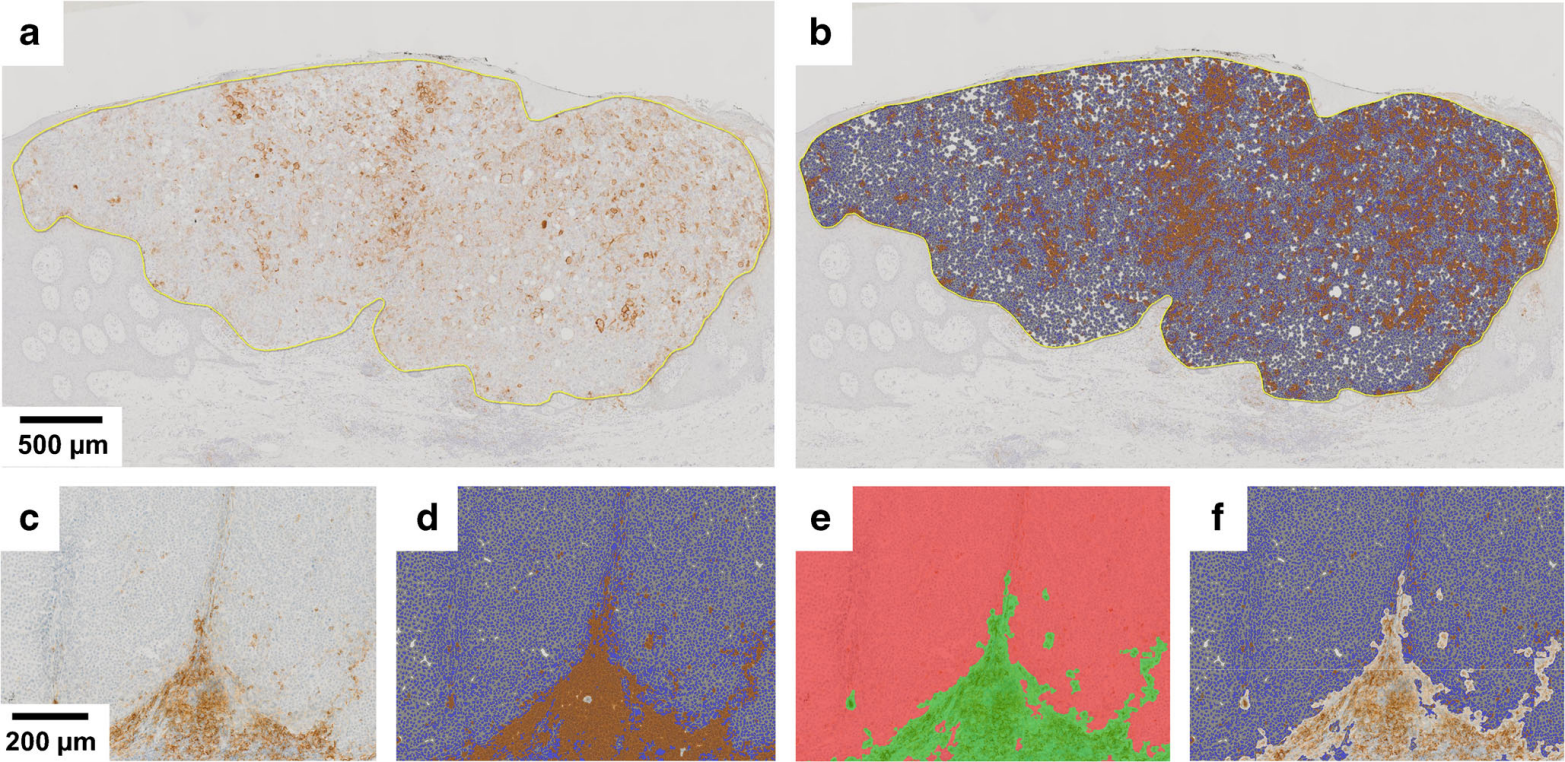
Assessment of PD-L1 expression by computational image analysis. **a** Malignant melanoma of the superficial spreading type stained for PD-L1 by IHC. Digital annotation of the tumor tissue is shown in yellow. Substantial marker heterogeneity and expression of PD-L1 in tumor-infiltrating inflammatory cells complicates conventional histopathological assessment of PD-L1 positivity. **b** Digital scoring of PD-L1 implemented on the HALO™ platform (Indica labs, Corrales, NM, USA). A total of 34.882 cells were detected by nuclear segmentation using the hematoxylin counterstain for nuclear seeding followed by cell/nuclear boundary detection and postprocessing according to pathologist-controlled cellular parameters, such as nuclear size, roundness, and optical density. Membranous reactivity for 3,3′-diaminobenzidine (DAB) is detected and analyzed according to pathologist-set positivity thresholds. PD-L1 negative stromal cells and normal squamous epithelium serve as on-slide negative controls. In this case, 11.802 PD-L1 positive cells were detected for a total of 33.8% positive cells within the annotation region. **c**–**d** PD-L1 reactivity in infiltrating immune cells can skew the assessment of PD-L1 expression in solid tumors with intrinsically low expression levels of PD-L1. **e**–**f** Machine learning algorithms trained on large sample sets to differentiate PD-L1 positive immune cells (green) from tumor cell populations (red) represent a powerful approach for tissue classification. Tissue classification is followed by cell-level analysis of DAB expression for the precise assessment of PD-L1 expression in tumor cells only

Digital scoring of PD-L1 can assist the pathologist to overcome these barriers by providing standardized metrics for biomarker assessment at single cell resolution across whole tissue sections [38, 44, 55] (Fig. 1a, b). A single tumor section can contain up to 10^6^–10^7^ cells, which can be rapidly and reproducibly scored using purpose-built image analysis algorithms [55]. Assessment of full tissue sections is a robust approach to account for tissue heterogeneity and to reliably measure PD-L1 expression at low levels [44, 55]. Fine-scale differences in biomarker compartmentalization can be captured at single cell resolution. ML technologies are beginning to be utilized in digital image analysis [40]. Pathologist input is critical for the creation of homogenous artificial intelligence training datasets that incorporate a sufficiently large number of true positive and negative staining examples as well as cases with unspecific staining and common technical artifacts. Expertly designed ML applications then have the potential to aid the human observer in assigning biomarker scores to defined cell populations based on morphological criteria and staining characteristics. For PD-L1 scoring, this approach can be particularly helpful to include or exclude PD-L1 expression in tumor infiltrating immune cells and tumor regions with non-specific, e.g., cytoplasmic staining (Fig. 1c–f)[44].

Standardized biomarker assessment protocols are essential to accelerate clinical development of immuno-oncology therapeutics [27]. Co-development of purpose-built computational pathology solutions in parallel to clinical trials may help to harmonize immunotherapy companion diagnostics, since image analysis algorithms can be easily shared and standardized across diagnostic labs. Alternatively, standardized biomarker testing may be conducted via telepathology at a central location. In addition, computational pathology applications can also increase the quality of biomarker assessment within an institution by using standardized tissue blocks and scanning procedures. While computational pathology will become a crucial tool in extracting quantitative information from digitized slides, digitization will also enable new applications. It will be possible to compare current cases with annotated phenotype libraries, hence effectively integrating a vast amount of knowledge into clinical decision-making [52]. Taken together, computational pathology methods are likely to become important tools for making the assessment of immune biomarkers more reproducible, robust, and reliable.

### Spatial analysis of tumor immune cell infiltration

The identification, localization, and spatial relationships of specific immune cells—before, during, or after therapy—have significant prognostic and predictive potential [10]. Both commercial and freely available open-source image analysis solutions are available to perform area-based quantification of immune cells by IHC or immunofluorescence (IF) for prognostic and predictive analyses [6, 25, 54]. This includes the enumeration of peri- and intratumoral CD3+ and CD8+ T cell populations per mm^2^ of tumor tissue to form the Immunoscore® (IS) as marketed by HalioDx [63]. This approach has shown independent prognostic value in CRC beyond usual risk factors and has strong potential to aid patient stratification in other solid tumors [41]. A worldwide consortium-based validation study in stage I–III CRC reported significantly longer recurrence free intervals in patients with high numbers of tumor-infiltrating T cells [63]. Recent data from stage III and stage IV CRC patients treated with adjuvant chemotherapy also indicate that the quantification of IS markers by image analysis has the potential to guide decisions on treatment duration and follow-up in advanced disease [57]. However, the predictive potential of the Immunoscore® for immunotherapy response beyond well-known predictors, such as DNA mismatch-repair (MMR) deficiency remains to be investigated [62].

**Fig. 2:**
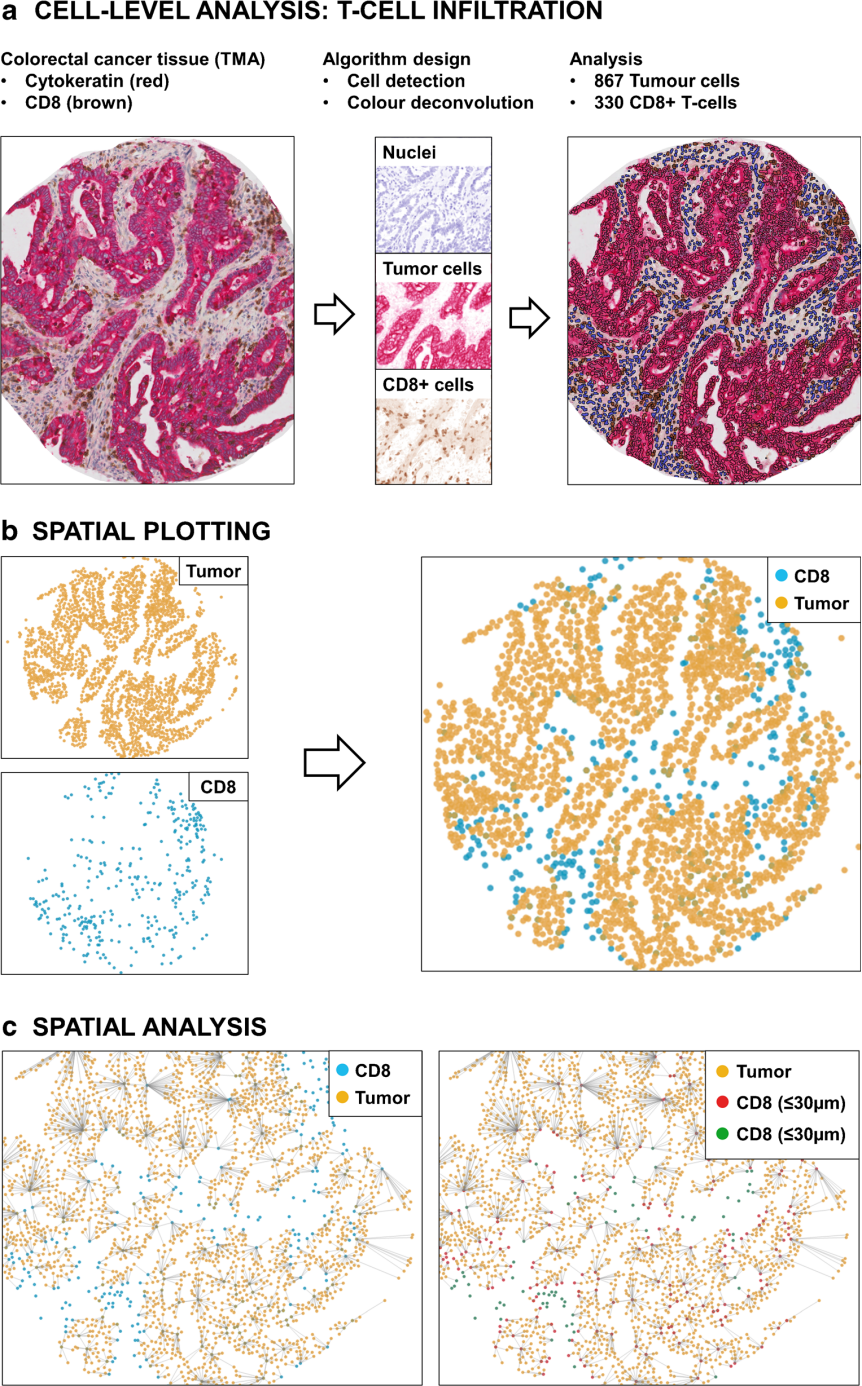
Spatial analysis of tumor immune cell infiltration. **a** Colorectal adenocarcinoma tissue microarray (TMA) spot stained for cytokeratin (Fast Red) and CD8+ T cells (DAB) with hematoxylin as a nuclear counterstain (left). Computational color deconvolution is performed for separate detection of cell nuclei, Fast Red, and DAB reaction products (middle), followed by nuclear segmentation and scoring of all cell populations (tumor cells: red; CD-8+ T cells: brown; marker negative cell nuclei: blue). A total of 1.623 cells were detected in this sample including 867 tumor cells, 330 CD8+ T cells, and 644 marker-negative cells. **b** Spatial plotting implemented on the HALO™ platform showing the localization of 867 cytokeratin positive tumor cells and 330 CD8+ T cells in this TMA spot. This allows to extract precise data on the relative distribution of T cells to the intraepithelial and stromal compartment in the tumor microenvironment. In this sample, 112 CD8+ T cells (or 33.9%) are localized to the intraepithelial compartment, while 218 CD8+ T cells (or 66.1%) localize to the tumor stroma. **c** Recording of the *x*-*y* coordinates in the tissue sample allows to define cell-cell relations by spatial analysis, such as the definition of nearest neighbor relationships between the tumor and CD8+ cell population (left) as well as the extraction of precise cell-cell distance measures (right)

Assessing immune cell infiltration is an important component of the “Cancer Immunogram” for patient stratification in future immunotherapy trials [10]. Initial studies have identified a broad association of T cell location at the tumor invasive margin and in nodal metastasis with response to ICI in melanoma patients [19, 79]. Modern digital image analysis techniques will enable a more detailed analysis: The exact coding of the *x*-*y* location of each individual marker positive cell on a histological slide for precise nearest neighbor and infiltration analyses is already feasible (Fig. 2a–c). This will allow to better understand the pathophysiology of tumor-host interaction, checkpoint molecule expression, and therapy effects in archival samples and preclinical models [8, 31]. Using these modern digital tools to record immune cell infiltrates over the course of therapy will empower data mining with patient characteristics, clinical response profiles, and genomic markers to build novel predictive indicators. Spatial profiling and compartmentalization studies can also be used to monitor and better understand immunotherapy associated adverse events which are commonly observed with ICI therapy [47].

### Multiplexing

To empower more accurate patient stratification for immunotherapy, histological analysis should aim for a simultaneous characterization of both immune and tumor-related pathways in a single tissue sample [10, 29, 73]. Multiplexed immunoprofiling is key to generate comprehensive biomarker datasets for correlation with clinical parameters [11, 80]. Digital image analysis is an effective tool to extract comprehensive information on biomarker expression levels, co-localization, and compartmentalization. Technical approaches include virtual multistaining by computational methods, simultaneous multimarker staining of single slides, and sequential staining and quenching protocols.

Image registration is a powerful approach to generate virtual multistains from serial sections using computational methods: Following sectioning, staining, and scanning, serial sections can be computationally aligned by automated or user guided approaches [82]. Digital image analysis is carried out and the results are used to generate virtual multimarker profiles. Standard manual or automated staining procedures can be used for visualization. Virtual multistaining also offers the unique opportunity of combining distinct histological methods, such as RNA in situ hybridization and IHC for multiparametric tissue profiling. This approach has been recently used to spatially correlate the expression of specific microRNAs, interferon-stimulated target genes, T cell infiltration, and the expression of cytotoxic effector molecules in the microenvironment of CRC [45]. Limitations include the requirement for multiple tissue sections, inevitable sectioning artifacts, and the need to perform complex image transformation procedures for precise image alignment.

Highly optimized multistaining panels in combination with purpose-built digital image analysis software can overcome these drawbacks. However, substantial investment into a multimodal staining platform and analysis software is required to tap into this technology. Examples of this approach include the Vectra® Polaris™ Automated Quantitative Pathology Imaging System introduced by PerkinElmer [35]. Using a single slide, this workflow allows to generate highly multiplexed IF panels to investigate six or more antigens in a strictly quantitative manner. Standardization is facilitated by commercially available marker panels, which may assist future clinical applications. A recent study by Mezheyeuski and colleagues elegantly illustrates the combination of the Vectra® approach with tissue microarray technology for a high-throughput profiling of the immune-environment in NSCLC samples [56]. By combining a tumor-specific marker with a panel of immune-cell associated antigens, the authors analyze both quantitative and spatial information of specific lymphocyte subpopulations with RNA-expression levels and prognosis.

Interesting alternatives to simultaneous multiplexed IF are sequential workflows consistent of staining, digitalization, inactivation of fluorescent dyes, and re-staining. Profiling of up to 100 IF biomarkers on a single tissue section is technically feasible [29, 70]. Exemplary applications include multiplexed fluorescence microscopy method (MxIF) [29, 73] and multiepitope-ligand cartography (MELC) [70]. Similar approaches have been tested and successfully applied for IHC protocols [66, 78]. The single channel outputs from each staining cycle can be easily merged into comprehensive expression maps for characterization of the tumor microenvironment using computational methods [78]. However, individual optimization of the staining protocols, tissue degradation with iterative staining cycles, process time, and standardization may be hurdles in applying this technology to large clinical trial sample sets.

CO-Detection by indEXing (CODEX) is among the most recent and innovative approaches for high-dimensional imaging of antibody-tagged epitopes in FFPE tissue [30]. CODEX staining uses antibodies labeled with unique DNA barcodes that are iteratively detected by in-situ polymerization with fluorescently labeled dNTP analogues. Tissue slides are stained in a single incubation step, followed by imaging cycles for the spatially resolved visualization of all antibody binding events. Pilot studies for have demonstrated the technical feasibility and power of this approach for deep profiling of immune tissue architecture [30]. Application to immunoprofiling of cancer tissues is a logical next step for detailed analysis of the microenvironment in correlation with clinical and molecular parameters. A decisive advantage of this approach is the possibility to image CODEX-labels on a standard three-color fluorescence microscope or scanner.

Combining spatial imaging approaches with mass spectrometry may serve to reach yet another dimension in precision profiling of the tumor microenvironment. Multiplexed ion beam imaging (MIBI) uses antibodies labeled with isotopically pure elemental metal reporters (mass tags) to simultaneously detect up to 100 targets on FFPE tissue sections [5]. Conventional histologic stains can be included in this panel, allowing precise reconstruction of virtual histology images with antibody expression data. Decisive technological advantages of MIBI include the stability of mass tag labels, a sensitivity exceeding chromogenic IHC by up to three log levels, and no spectral overlap between individual labels [5]. Recent studies have demonstrated the technical feasibility to integrate RNAscope-based metal in situ hybridization with MIBI for multiparametric profiling [71]. Purpose-built digital image analysis methods are available to reconstruct virtual histology images and explore cell phenotypes, spatial interaction, and morphological structures [69]. Further technological advances using dissociated tissue samples allow to perform single cell proteomic analysis using a mass spectrometry-flow cytometry hybrid device, the so-called “CyTOF” [9]. However, substantial investment and expertise in mass spectrometry are necessary to access this technology.

### Morpho-molecular integration

The recently proposed “Cancer Immunogram” suggests seven categories including tumor foreignness, general immune status, immune cell infiltration, absence of checkpoints, absence of soluble inhibitors, absence of inhibitory tumor metabolism, and tumor sensitivity to immune effectors as the most important predictors of immunotherapy response [10]. As such, the “Cancer Immunogram” is highly integrative and includes both tumor- and immune-related parameters assessed with both molecular and image-based methods for individualized prediction of immunotherapy response. By providing continuous data on tissue-based parameters, such as immune cell infiltration and expression of immune checkpoints, computational pathology methods are ideally suited for data integration with molecular parameters.

Tumors with a high mutational load frequently exhibit an immunologically activated phenotype [4]. Recent study has linked defects in DNA mismatch repair to high frequencies of neoantigens and immunotherapy response in advanced solid tumors [49, 62]. However, the correlation between genomic parameters and immunogenicity is far from linear [53]. In some solid tumors, such as melanoma, a lack of association of neoantigen type and frequency with baseline immune activation has been elegantly demonstrated [74]. In these tumors, strong regulatory T cell infiltration may curb the anti-tumoral immune response [75]. Other known mechanisms that may impact tumor antigenicity are defects in antigen-presentation and overexpression of immunosuppressive molecules, such as CD47, PD-L1, and indolamine-2,3-dioxygenase [42, 43, 75]. These image-based features can be reliably captured by computational image analysis methods for in-depth profiling of the tumor microenvironment.

Incorporating digital information on immune cell infiltration with molecular data is a powerful approach to inform the “Cancer Immunogram.” Conde and colleagues recently provided a proof of principle for the association of specific genetic alterations with defined immunophenotypes by combining digital assessment of CD8+ T cell infiltration in lung squamous-cell carcinoma with manual scoring of PD-L1 and targeted next generation sequencing [16]. Joining digital and molecular pathology also facilitates the development of novel assays to predict immunotherapy response. We have recently developed and validated a computational pathology assay that identifies specific PD1-positive subpopulations in NSCLC as a powerful predictive indicator of response to ICI treatment [76]. Methodologically, this assay was driven by morpho-molecular integration of RNA sequencing data with tissue-based methods. Computational image analysis was a key tool in this process, driving the development of a standardized algorithm for the detection of tumor-infiltrating lymphocyte populations with uniquely high expression levels of PD1 protein (PD1^T^ lymphocytes). PD1^T^ cells were reproducibly detected and quantified in pretreatment biopsies of lung cancer patients and correlated strongly with treatment response to ICI in two independent clinical cohorts. This translational approach highlights how digital image analysis can represent a powerful companion diagnostic for cancer immunotherapy applications.

### Machine learning and artificial intelligence

ML will transform the field of immuno-oncology. We expect that ML will drive a paradigm shift in the way data are collected and analyzed to discover new prognostic markers or to construct more rigorous risk classification to empower stratified medicine. ML is essentially a set of computer algorithms that learn generic rules to perform any given task directly from data, without requirements of predefined knowledge or domain expertise. This, in many ways, is similar to how pathologists have previously gained knowledge and expertise through continuous practice that has led to new diagnostic classifications or prognostic factors in the clinical routine.

Since ML is heavily data-driven, it enables a means to derive unbiased statistics from data. Accurate and continuous variables are more informative and can provide more biologically relevant information than the semi-quantitative scores presently implemented in diagnostic practice. Although several classical regression models exist to facilitate the discovery process of predictive or prognostic factors, the power of ML surpasses that of traditional tools when data is large and complex. ML can crunch through a vast amount of data and learn complex relationships between parameters and outcomes without the need to prespecify the relationships as normally required in traditional regression models. Nonetheless, data-driven approaches have critical downsides if not used with caution. ML can learn meaningless, biologically uninterpretable features that correlate to outcomes if the data has not been carefully preprocessed to remove any spurious features. Training datasets must have been generated in a highly standardized manner; otherwise, AI algorithms are likely to “misinterpret” sampling differences and artifacts between sets as distinctive biological characteristics. This phenomenon is known as “garbage in, garbage out.” Expert pathologist input in the training of image analysis algorithms is therefore of critical importance. Furthermore, ML tends to “overfit” to discovery data which results in an over-optimistic estimate of the performance of a model, while, in fact, it cannot be generalized well to new unseen datasets. It is, therefore, necessary that ML models are rigorously validated on new datasets that are independent of those used during the model development. Originating from the field of computer vision, deep convolutional neural network (DCNN) is a ML approach that is specialized in image analysis tasks [46, 50, 51]. In recent years, DCNN has been successfully applied to key applications in diagnostic pathology, including cell classification [72], cell enumeration [85], tumor grading [23], cancer diagnosis [22], and cancer prognostication [58].

#### Application of machine learning in immuno-oncology: pattern recognition

In immuno-oncology, ML as a pattern recognition tool enables an accurate and reproducible means for the unbiased assessment of regularities in the expression of immunohistochemical markers, tumor morphology, and the spatial distribution of tumor infiltrating lymphocytes (TILs). The ability of ML tools to detect key features in complex immunophenotypic datasets underlines their potential importance for the development of novel predictive models in cancer research. Initial studies underline the potential of ML methods for clinical translation:

Yuan et al. computationally profiled cells of triple-negative breast cancer cohorts on H&E-stained sections and were able to uncover three categories of lymphocytes (intra-tumor, adjacent-tumor, and distal-tumor) with an unsupervised clustering method based on the proximities of lymphocytes to tumor cells [87]. Interestingly, the ratio between the total number of intratumoural lymphocytes and the number of cancer cells was strongly associated with disease-specific survival and strongly correlated with the expression of CTLA-4, a known immunotherapy target. Saltz et al. employed a DCNN model to determine the probability of TIL infiltration for every small region of H&E stained sections [68]. This resulted in a map which indicates the degree of local lymphocyte infiltration. Unsupervised clustering methods were employed to group small TIL regions into spatially coherent structures [28]. The resulting clustering patterns were characterized using parameters related to cluster size and shape. Importantly, the covariates summarizing complex characteristics of clusters were found to be associated with overall survival in various cancer groups, illustrating the potential of ML methodologies applied to standard H&E slides for the development of biomarkers in immuno-oncology.

Heindl et al. demonstrated that quantifying the number of spatial clusters or hotspots of immune cells and cancer cells is prognostic in ER+ breast cancer using a fully automated H&E stained image analysis algorithm [32]. The spatial clustering is identified as an area in which the number of cells of interest is greater than expected by chance given the distribution of the cells on the whole tissue section. The increased immune spatial clustering is associated with poor prognosis and has a level of prognostic significance on par with the IHC test [18] and the Oncotype DX 21-gene recurrence score [64]. Interestingly, immune scores which are related to the ratios of distinct categories of lymphocytes to the total number of cancer cells did not provide prognostic information in this study. This illustrates the importance of ML tools to detect novel morphologic features that provide prognostic information beyond established classifications.

#### Application of machine learning in Immuno-oncology: survival analysis

In medical research, survival analysis is a traditional means to assess the prognostic significance of each candidate covariate. The most widely used survival model is the Cox proportional hazards model. It assumes that the risk of an event of interest to occur (failure) is not time-dependent, is determined by a linear combination of all covariates, and might also include interaction terms between the covariates. This somewhat too simplistic assumption may not be able to capture the precise effects of the observed medical covariates on the risk of failure. This is particularly true in immuno-oncology where variable effects of immune infiltrates and immune-related gene signatures are observed depending on the tumor type and host immune status [7]. DNN applied to survival analysis could provide a better way to model complex nonlinear relationships among prognostic factors that better fit to the survival data.

It has been shown that DNN performs better than the Cox proportional hazard model in various medical applications [24, 58, 86]. Katzman et al. investigated the risk associated with treatment choices and demonstrated that their DeepSurv system could provide treatment recommendations that increase the median survival time of patients [37]. Yousefi et al. used their SurvivalNet to analyze large-scale genomic profiles obtained from the TCGA database [14, 86]. They have demonstrated the robustness of the model across different cancer types even if the number of input covariates is sometimes considerably larger than the sample size. Moreover, the model also allows the interpretation of the prognostic significance of individual covariates based on their contributions to overall risk. The same model has been extended to allow integration between histology image and genomic data in a unified framework [58]. Likewise, specific methylome signatures queried by ML were shown to be suitable for prediction of response to immune-checkpoint inhibitors. In analogy to image analysis, this modality takes both neoplastic and reactive cells into account [21]. These results suggest the potential of deep learning as a discovery tool to provide insight into the biology of immuno-oncology.

### Challenges

Regulatory issues in the use of digital pathology devices have been broadly recognized by national and international bodies. Pioneering companies have recently received CE certification for routine pathology applications in the European Union under the In vitro *diagnostic medical devices* directive [1]. Comprehensive, non-binding recommendations have been issued by the Food and Drug Administration (FDA) and the College of American pathologists (CAP) [3, 65]. These guidelines highlight two important aspects of testing emerging biomarkers for immunotherapy by computational pathology methods: First, the quality and reliability of the imaging system as recommended by the FDA and mandated by the CE certification process, and second, the consistency of diagnoses made by pathologists using digital systems as advised by the CAP [3, 65]. Both are of essential importance in the design of reliable computational pathology workflows to inform clinical decision making.

Concerns have been raised that inaccurate study design, statistical analysis, and reporting of research lead to a significant waste of research funding and inaccurate scientific conclusions [36]. A major challenge inherent to the use of digital image analysis is the substantial expansion in the number of variables. Although it may seem attractive from a scientific perspective to extract as much information as possible from a limited amount of tissue, it is important to recognize that big data mining inherently increases the level of statistical noise. Robust statistical considerations are required to avoid multiple testing problems and misleading conclusions. As with any diagnostic tool, digital image analysis is therefore critically dependent on scientific rigor, reliable documentation, consequent quality control, and adequate training of the pathology workforce [48]. Unsolved problems also concern the standardization of the preanalytical steps before the imaging and analysis of tissue slides. A perfect example is Ki-67 staining, which has proven difficult to harmonize between labs despite being one of the most frequently performed assay of its kind [26]. The same applies to the majority of immunohistochemical stains, in particular those where expression levels matter.

### Conclusions

Advanced multiparametric imaging applications and ML have the potential to translate our evolving understanding of tumor-host interaction into better patient stratification and new treatment strategies. Computational pathology will help to derive complete, standardized, and reproducible datasets to facilitate the individualized prediction of immunotherapy response. Upskilling the pathology workforce through professional education and the recognition of computational pathology by professional societies will be essential to meet future clinical demands for optimal patient care.
